# Supplementary material for: Genetic History of the Altai Breed Horses: From Ancient Times to Modernity
Source: Genes (Basel). 2023 Jul 26;14(8):1523. doi: 10.3390/genes14081523 (PMC10454587; doi:10.3390/genes14081523)
Supplement: Supplementary file 1 [file genes-14-01523-s001.zip › File S1. AMOVA hierarchical molecular variance distribution..pdf]

**Table S5.** AMOVA hierarchical molecular variance distribution (difference between more and less high-altitude groups).

| **Source of variation** | **d.f. (degrees of freedom)** | **Variance component** | **Variance (%)** | **Fixation index** | ***P-value*** |
|---|---|---|---|---|---|
| Between united groups[1] | 1 | 0.13958 Va[2] | 5.71 | $F_{CT}$ = 0.05711 | 0.33822 ± 0.01681 |
| Between groups within a priori formed united groups | 1 | 0.04407 Vb[2] | 1.80 | $F_{SC}$ = 0.01912 | 0.09971 ± 0.00834 |
| Within groups | 125 | 2.26056 Vc[2] | 92.49 | $F_{ST}$ = 0.07514 | 0.00684 ± 0.00271 |

[1]United group 1 — Kosh-Agach group, united group 2 — Ulagan groups (“mixed” + gene pool).
[2]Variance component name.
“$F_{CT}$” — Va/(Va+Vb+Vc), the proportion of variation among groups.
“$F_{SC}$” — Vb/(Vb+Vc), the proportion of variation among groups within a priori formed united groups.
“$F_{ST}$” — Va+Vb/(Va+Vb+Vc), the proportion of variation within groups.

**Table S6.** AMOVA hierarchical molecular variance distribution (difference between gene pool and “mixed” groups).

| **Source of variation** | **d.f. (degrees of freedom)** | **Variance component** | **Variance (%)** | **Fixation index** | ***P-value*** |
|---|---|---|---|---|---|
| Between united groups[1] | 1 | - 0.13427 Va[2] | - 5.71 | $F_{CT}$ = - 0.05708 | 1.00000 ± 0.00000 |
| Between groups within a priori formed united groups | 1 | 0.22599 Vb[2] | 9.61 | $F_{SC}$ = 0.09088 | 0.01857 ± 0.00437 |
| Within groups | 125 | 2.26056 Vc[2] | 96.10 | $F_{ST}$ = 0.03899 | 0.00978 ± 0.00326 |

[1]United group 1 — gene pool Ulagan group, united group 2 — “mixed” Ulagan group + “mixed” Kosh-Agach group.
[2]Variance component name.
“$F_{CT}$” — Va/(Va+Vb+Vc), the proportion of variation among groups.
“$F_{SC}$” — Vb/(Vb+Vc), the proportion of variation among groups within a priori formed united groups.
“$F_{ST}$” — Va+Vb/(Va+Vb+Vc), the proportion of variation within groups.

**Table S7.** Hierarchical molecular variance distribution according to AMOVA results (difference between the “mixed” Ulagan group and other studied groups).

| **Source of variation** | **d.f. (degrees of freedom)** | **Variance component** | **Variance (%)** | **Fixation index** | ***P-value*** |
|---|---|---|---|---|---|
| Between united groups[1] | 1 | - 0.09135 Va[2] | - 3.91 | $F_{CT}$ = - 0.03907 | 0.66080 ± 0.01542 |
| Between groups within a priori formed united groups | 1 | 0.16849 Vb[2] | 7.21 | $F_{SC}$ = 0.06936 | 0.01466 ± 0.00340 |

| | | | | | |
|---|---|---|---|---|---|
| Within groups | 125 | 2.26056 Vc[2] | 96.70 | $F_{ST}$ = 0.03300 | 0.00587 ± 0.00219 |

[1]United group 1 — "mixed" Ulagan group, united group 2 — gene pool Ulagan group + "mixed" Kosh-Agach group.
[2]Variance component name.
"$F_{CT}$" — Va/(Va+Vb+Vc), the proportion of variation among groups.
"$F_{SC}$" — Vb/(Vb+Vc), the proportion of variation among groups within a priori formed united groups.
"$F_{ST}$" — Va+Vb/(Va+Vb+Vc), the proportion of variation within groups.
