# Supplementary material for: Genetic History of the Altai Breed Horses: From Ancient Times to Modernity
Source: Genes (Basel). 2023 Jul 26;14(8):1523. doi: 10.3390/genes14081523 (PMC10454587; doi:10.3390/genes14081523)
Supplement: Supplementary file 1 [file genes-14-01523-s001.zip › Table S1. Information about the samples of ancient and medieval horses of Altai and Mongolia.pdf]

Table S1. Information about the samples of ancient and medieval horses of Altai and Mongolia and the results of the analysis of the mitogenome control region of these horses.

Name of sample	Geographic origin of sample	GPS coordinates of archaeological site	Name of archaeological culture	Material type of sample	Nucleotide polymorphisms not in the mutation hotspots (15495–15720 bp fragment)	Nucleotide polymorphisms in the mutation hotspots (15495–15720 bp fragment)	Mitotype	Sequence accession № (GenBank)
DC	Altai	51.397581, 84.676206	– (Paleolithic)	Tooth	495C, 497T, 526C, 536C, 566C, 602T, 709T, 720A	597G, 495C, 526C, 566C, 602T, 649G, 720A	X-11+497+536+709-649 (~ X11-group)	OR001883
Ak2-4	Altai	49.299718, 87.562500	Biiken	Trochanter	495C, 602T, 703C, 720A	585A, 597G	K	OR001888
Ak2-5	Altai	49.299718, 87.562500	Biiken	Trochanter	495C	–	D2	OR001887
Ak2-6	Altai	49.299718, 87.562500	Biiken	Trochanter	495C, 602T, 703C, 720A, 740G	604A	K2	OR001886
Ak1-1	Altai	49.285356, 87.535506	Pazyryk	Outer splint	495C, 538G, 602T, 709T, 720A	585A, 650G	I	OR001884
Ver1-1	Altai	49.200060, 87.356356	Pazyryk	Femur	495C, 601C, 602T, 703C, 720A	–	F+703, K+601	OR001885
Ber2	Altai	51.253151, 86.070825	Pazyryk	Tooth	494C, 495C, 496G, 534T, 603C, 649G, 720A	585A	X2b	OR001898
Bi3	Altai	51.282068, 86.059524	Pazyryk	Carpus	495C, 521A, 596G, 602T, 720A	–	G1	OR001899
Bi4	Altai	51.165778, 86.145278	Turkic	Pars petrosa of the temporal bone	495C, 538G, 602T, 709T, 720A	597G, 650G	I	OR001900
Ush-7	Mongolia	49.655567, 99.927100	Khereksur and Deer Stone	Vertebrae cervicales	495C, 720A	–	D	OR001895

Ush-8	Mongolia	49.655567, 99.927100	Khereksur and Deer Stone	Vertebrae cervicales	494C, 495C, 496G, 534T, 603C, 649G, 720A	585A	X2b	OR001896
Ush-10	Mongolia	49.655567, 99.927100	Khereksur and Deer Stone	Vertebrae cervicales	495C, 602T, 709T, 720A	597G, 650G	A+709	OR001897
Gan-1	Mongolia	49.572897, 103.251933	Khereksur and Deer Stone	Pars petrosa of the temporal bone	495C, 602T, 617C, 659C, 720A	–	B1	OR001889
Gan-3	Mongolia	49.572897, 103.251933	Khereksur and Deer Stone	Tooth	495C, 602T, 617C, 659C, 720A	–	B1	OR001890
Gan-11	Mongolia	49.573011, 103.255725	Khereksur and Deer Stone	Tooth	495C, 542T, 602T, 635T, 666A, 703C, 720A	585A, 597G, 650G	X3c1	OR001891
Gan-14	Mongolia	49.575922, 103.250372	Khereksur and Deer Stone	Tooth	494C, 495C, 496G, 534T, 602T, 603C, 649G, 720A	585A, 604A	X2	OR001892
Gan-18	Mongolia	49.575922, 103.250372	Khereksur and Deer Stone	Tooth	494C, 495C, 496G, 534T, 603C, 649G, 720A	585A, 604A	X2b	OR001893
Er-1	Mongolia	49.541831, 103.259603	Xiongnu	Tooth	495C, 542T, 602T, 635T, 666A, 703C, 720A	585A, 597G, 650G	X3c1	OR001894

"number XXX" — nucleotide position in the *Equus caballus* mitogenome (NC_001640.1) after 15,000 bp.
