# Supplementary material for: Genetic History of the Altai Breed Horses: From Ancient Times to Modernity
Source: Genes (Basel). 2023 Jul 26;14(8):1523. doi: 10.3390/genes14081523 (PMC10454587; doi:10.3390/genes14081523)
Supplement: Supplementary file 1 [file genes-14-01523-s001.zip › Table S2. Haplogroup frequencies of the modern horse groups studied here.pdf]

Table S2. Haplogroup frequencies of the modern horse groups studied here (source data for the PCA).

Name of group	Number of samples	Mitogenome haplogroup frequencies															
		**A**	**B**	**C**	**D**	**D3**	**E**	**F**	**G**	**I**	**K**	**K3**	**X2**	**X3**	**X4**	**X5**	**X7**
MMP	231	12.55	6.93	0.43	9.52	4.33	0.43	3.03	1.30	3.03	20.35	6.93	17.75	11.26	0.87	0.43	0.87
MSP	52	18.75	6.25	0.00	8.33	12.50	0.00	0.00	0.00	2.08	10.42	8.33	20.83	19.23	0.00	0.00	0.00
MAUG	81	6.17	4.94	0.00	0.00	0.00	0.00	7.41	0.00	0.00	20.99	30.86	22.22	4.94	2.47	0.00	0.00
MAUM	29	10.34	0.00	0.00	10.34	0.00	0.00	0.00	0.00	10.34	24.14	13.79	10.34	6.90	13.79	0.00	0.00
MAKM	18	0.00	22.22	0.00	0.00	0.00	0.00	5.56	0.00	0.00	11.11	11.11	38.89	0.00	11.11	0.00	0.00
