# Supplementary material for: Genetic History of the Altai Breed Horses: From Ancient Times to Modernity
Source: Genes (Basel). 2023 Jul 26;14(8):1523. doi: 10.3390/genes14081523 (PMC10454587; doi:10.3390/genes14081523)
Supplement: Supplementary file 1 [file genes-14-01523-s001.zip › Table S3. Identified haplotypes of modern Altai horses studied here.pdf]

Table S3. Identified haplotypes of modern Altai horses studied here.

№	Name of sample	Name of group	Gender of horse	Nucleotide variants in the 15495–15720 bp horse mitogenome fragment (NC_001640.1)	Nucleotide variants in the horse mitogenome control region mutation hotspots	Mitogenome control region haplotype	Sequence accession № (GenBank)
1	T1_1.1	gene pool Ulagan	stallion	T495C, C602T, T652C, G720A	A650G	A + 652	KT827401.1
2	T1_1.2	gene pool Ulagan	mare	T495C, C602T, T703C, G720A, A740G	G585A, G604A	K2	KT827420.1
3	T1_1.3	gene pool Ulagan	stallion	T495C, C602T, T703C, G720A, A740G	G585A, G604A	K2	KT827421.1
4	T1_1.5	gene pool Ulagan	stallion	T495C, C602T, T703C, G720A, G726A, A740G	A597G, G604A	K2b	KT827428.1
5	T1_1.6	gene pool Ulagan	mare	T495C, C602T, T703C, G720A	A597G, G604A	K	KT827429.1
6	T1_2.1	gene pool Ulagan	mare	T494C, T495C, A496G, C534T, C602T, T603C, A649G, G720A	G585A	X2	KT827459.1
7	T1_2.2	gene pool Ulagan	mare	T495C, C542T, C602T, C635T, G666A, T703C, G720A	G585A, A597G, A650G	X3c1	KT827476.1
8	T1_2.3	gene pool Ulagan	mare	T495C, C584T, T601C, C602T, G720A	G585A	F + 584	KT827415.1
9	T1_3.1	gene pool Ulagan	mare	T494C, T495C, A496G, C534T, T603C, A649G, G720A	G585A	X2b	KT827464.1
10	T1_3.2	gene pool Ulagan	mare	T495C, C542T, C602T, C635T, G666A, T703C, G720A	G585A, A597G, A650G	X3c1	KT827477.1
11	T1_3.3	gene pool Ulagan	mare	T495C, C602T, A667G, T703C, G720A	A597G, G604A	K3	KT827434.1
12	T1_3.4	gene pool Ulagan	mare	T494C, T495C, A496G, C534T, T603C, A649G, G720A	G585A	X2b	KT827465.1
13	T1_4.2	gene pool Ulagan	mare	T494C, T495C, A496G, C534T, T603C, A649G, G720A	G585A	X2b	KT827466.1
14	T1_4.3	gene pool Ulagan	mare	T495C, C602T, A667G, T703C, G720A	A597G, G604A	K3	KT827435.1
15	T1_4.4	gene pool Ulagan	stallion	T494C, T495C, A496G, C534T, T603C, A649G, G720A	G585A	X2b	KT827467.1
16	T1_5.1	gene pool Ulagan	mare	T494C, T495C, A496G, C534T, T603C, A649G, G720A	G585A	X2b	KT827468.1

17	T1_5.2	gene pool Ulagan	mare	T495C, T526C, A540G, C602T, A649G, C718T, G720A	G585A	X4a	KT827480.1
18	T1_5.4	gene pool Ulagan	mare	T495C, C602T, T703C, G720A	G585A, A597G	K	KT827416.1
19	T1_5.5	gene pool Ulagan	mare	T495C, C602T, T703C, G720A	G585A, A597G	K	KT827417.1
20	T1_6.2	gene pool Ulagan	stallion	T495C, T526C, A540G, C602T, A649G, C718T, G720A	G585A	X4a	KT827481.1
21	T2_K99	gene pool Ulagan	stallion	T495C, C602T, T703C, G720A, A740G	G604A	K2	KT827425.1
22	T2_K100	gene pool Ulagan	mare	T494C, T495C, A496G, C534T, T603C, A649G, G720A	G585A	X2b	KT827469.1
23	T2_K102	gene pool Ulagan	mare	T495C, C602T, T703C, G720A	G585A, A597G	K	KT827418.1
24	T2_K104	gene pool Ulagan	mare	T494C, T495C, A496G, C534T, C602T, T603C, A649G, G720A	–	X2	KT827458.1
25	T2_K105	gene pool Ulagan	stallion	T495C, C602T, A667G, T703C, G720A	G585A, A597G, G604A	K3	KT827447.1
26	T2_K108	gene pool Ulagan	stallion	T495C, C602T, A667G, T703C, G720A	A597G	K3	KT827433.1
27	T2_K112	gene pool Ulagan	mare	T495C, C602T, T703C, G720A, A740G	G585A, G604A	K2	KT827423.1
28	T2_K113	gene pool Ulagan	stallion	T495C, C602T, T652C, G720A	A650G	A + 652	KT827402.1
29	T2_K114	gene pool Ulagan	stallion	T495C, C602T, T652C, G720A	A650G	A + 652	KT827403.1
30	T2_K117	gene pool Ulagan	mare	T495C, C602T, C635T, A667G, T703C, G720A	A597G, G604A	K3a	KT827454.1
31	T2_K119	gene pool Ulagan	mare	T495C, C602T, C635T, A667G, T703C, G720A	A597G, G604A	K3a	KT827455.1
32	T2_K124	gene pool Ulagan	stallion	T495C, C602T, T652C, G720A	A650G	A + 652	KT827404.1
33	T2_K126	gene pool Ulagan	mare	T494C, T495C, A496G, C534T, C602T, T603C, A649G, G720A	G585A	X2	KT827460.1
34	T3_1.1	gene pool Ulagan	mare	T495C, C602T, A667G, T703C, G720A	G585A, A597G, G604A	K3	KT827448.1
35	T3_1.3	gene pool Ulagan	mare	T495C, C602T, A667G, T703C, G720A	A597G, G604A	K3	KT827436.1
36	T3_1.5	gene pool	mare	T495C, C602T, A667G, T703C, G720A	G585A, A597G,	K3	KT827449.1

		Ulagan			G604A		
37	T3_1.6	gene pool Ulagan	stallion	T495C, C602T, A667G, T703C, G720A	G585A, A597G, G604A	K3	KT827450.1
38	T3_3.2	gene pool Ulagan	mare	T494C, T495C, A496G, C534T, T603C, A649G, G720A	G585A	X2b	KT827470.1
39	T3_3.5	gene pool Ulagan	stallion	T495C, C602T, C635T, A667G, T703C, G720A	A597G, G604A	K3a	KT827456.1
40	T3_4.1	gene pool Ulagan	mare	T494C, T495C, A496G, C534T, T603C, A649G, G720A	–	F	KT827410.1
41	T3_4.4	gene pool Ulagan	mare	T495C, C602T, A667G, T703C, G720A	A597G, G604A	K3	KT827437.1
42	T3_4.5	gene pool Ulagan	stallion	T494C, T495C, A496G, C534T, T603C, A649G, G720A	G585A	X2b	KT827471.1
43	T3_5.1	gene pool Ulagan	mare	T494C, T495C, A496G, C534T, T603C, A649G, G720A	–	F	KT827411.1
44	T3_5.2	gene pool Ulagan	mare	T494C, T495C, A496G, C534T, C602T, T603C, A649G, G720A	G585A	X2	KT827461.1
45	T3_5.3	gene pool Ulagan	stallion	T494C, T495C, A496G, C534T, T603C, A649G, G720A	G585A	X2b	KT827472.1
46	T3_5.4	gene pool Ulagan	mare	T495C, C602T, A667G, T703C, G720A	A597G, G604A	K3	KT827438.1
47	T3_5.5	gene pool Ulagan	mare	T495C, C602T, C635T, A667G, T703C, G720A	A597G, G604A	K3a	KT827457.1
48	T3_5.6	gene pool Ulagan	mare	T495C, C602T, A667G, T703C, G720A	A597G, G604A	K3	KT827439.1
49	T3_6.1	gene pool Ulagan	mare	T495C, C602T, A667G, T703C, G720A	A597G, G604A	K3	KT827440.1
50	T3_6.2	gene pool Ulagan	mare	T494C, T495C, A496G, C534T, T603C, A649G, G720A	G585A	X2b	KT827473.1
51	T3_6.3	gene pool Ulagan	mare	T495C, C602T, T703C, G720A, G726A, A740G	A597G, G604A	K2b	KT827430.1
52	T3_6.4	gene pool Ulagan	mare	T495C, C602T, T703C, G720A, G726A, A740G	A597G, G604A	K2b	KT827431.1
53	T3_6.5	gene pool Ulagan	mare	T495C, C602T, A667G, T703C, G720A	G585A, A597G, G604A	K3	KT827451.1
54	T3_7.1	gene pool Ulagan	mare	T495C, C602T, T703C, G720A, G726A, A740G	A597G, G604A	K2b	KT827432.1
55	T3_7.2	gene pool Ulagan	mare	T495C, C602T, T703C, G720A, A740G	G585A, G604A	K2	KT827424.1

56	T4_1.1	gene pool Ulagan	stallion	T495C, 601C, 602T, 720A	–	F	KT827412.1
57	T4_1.2	gene pool Ulagan	mare	T495C, C602T, T703C, G720A, A740G	G604A	K2	KT827426.1
58	T4_1.3	gene pool Ulagan	stallion	T495C, C602T, T703C, G720A, A740G	G604A	K2	KT827427.1
59	T4_1.4	gene pool Ulagan	stallion	T494C, T495C, A496G, C534T, C602T, T603C, A649G, G720A	G585A	X2	KT827462.1
60	T4_2.2	gene pool Ulagan	mare	T495C, C602T, A667G, T703C, G720A	A597G, G604A	K3	KT827441.1
61	T4_2.3	gene pool Ulagan	mare	T495C, C602T, A667G, T703C, G720A	A597G, G604A	K3	KT827442.1
62	T4_2.4	gene pool Ulagan	mare	T495C, C602T, A667G, T703C, G720A	A597G, G604A	K3	KT827443.1
63	T4_3.1	gene pool Ulagan	mare	T495C, C602T, T617C, T659C, G720A	–	B1	KT827406.1
64	T4_3.3	gene pool Ulagan	mare	T495C, C602T, T617C, T659C, G720A	–	B1	KT827407.1
65	T4_4.1	gene pool Ulagan	mare	T495C, C602T, T652C, G720A	A650G	A + 652	KT827405.1
66	T4_4.3	gene pool Ulagan	mare	T494C, T495C, A496G, C534T, C602T, T603C, A649G, G720A	G585A	X2	KT827463.1
67	T4_5.1	gene pool Ulagan	mare	T495C, C602T, T617C, T659C, G720A	–	B1	KT827408.1
68	T4_5.2	gene pool Ulagan	mare	T495C, C602T, A667G, T703C, G720A	G585A, A597G, G604A	K3	KT827452.1
69	T4_5.3	gene pool Ulagan	stallion	T495C, C602T, T703C, G720A, A740G	G585A, G604A	K2	KT827422.1
70	T4_5.4	gene pool Ulagan	stallion	T495C, C602T, T617C, T659C, G720A	–	B1	KT827409.1
71	T4_5.5	gene pool Ulagan	mare	T495C, 601C, 602T, 720A	–	F	KT827413.1
72	T4_6.1	gene pool Ulagan	mare	T495C, C542T, C602T, C635T, G666A, T703C, G720A	G585A, A597G, A650G	X3c1	KT827478.1
73	T4_6.2	gene pool Ulagan	mare	T495C, T601C, C602T, G720A	–	F	KT827414.1
74	T4_6.3	gene pool Ulagan	stallion	T495C, C542T, C602T, C635T, G666A, T703C, G720A	G585A, A597G, A650G	X3c1	KT827479.1
75	T4_6.4	gene pool	mare	T495C, C602T, A667G, T703C, G720A	A597G, G604A	K3	KT827444.1

		Ulagan					
76	T4_6.5	gene pool Ulagan	stallion	T495C, C602T, A667G, T703C, G720A	G585A, A597G, G604A	K3	KT827453.1
77	T4_7.1	gene pool Ulagan	mare	T494C, T495C, A496G, C534T, T603C, A649G, G720A	G585A	X2b	KT827474.1
78	T4_7.2	gene pool Ulagan	mare	T495C, C602T, A667G, T703C, G720A	A597G, G604A	K3	KT827445.1
79	T4_7.3	gene pool Ulagan	mare	T494C, T495C, A496G, C534T, T603C, A649G, G720A	G585A	X2b	KT827475.1
80	T4_7.4	gene pool Ulagan	mare	T495C, C602T, A667G, T703C, G720A	A597G, G604A	K3	KT827446.1
81	T4_7.5	gene pool Ulagan	mare	T495C, C602T, T703C, G720A	G585A, A597G	K	KT827419.1
82	T5_1.1	“mixed” Ulagan	mare	T495C, C602T, T703C, G720A	G585A, A597G	K	KT808297.1
83	T5_1.2	“mixed” Ulagan	stallion	T495C, C602T, T703C, G720A	G585A, A597G	K	KT808298.1
84	T5_2.1	“mixed” Ulagan	stallion	T495C, T526C, A540G, C602T, A649G, C718T, G720A	G585A	X4a	KT808313.1
85	T5_3.1	“mixed” Ulagan	–	T494C, T495C, A496G, C528T, C534T, C602T, T603C, A649G, G720A	–	X2 + 528	KT808310.1
86	T5_3.2	“mixed” Ulagan	mare	T495C, C602T, T703C, G720A, A740G	G585A, G604A	K2	KT808299.1
87	T5_4.1	“mixed” Ulagan	mare	T495C, C602T, A667G, T703C, G720A	A597G, G604A	K3	KT808304.1
88	T5_4.2	“mixed” Ulagan	stallion	T495C, C602T, A667G, T703C, G720A	A597G, G604A	K3	KT808305.1
89	T6_1.1	“mixed” Ulagan	mare	T495C, A538G, C602T, C709T, G720A	G585A, A650G	I	KT808294.1
90	T6_2.1	“mixed” Ulagan	mare	T495C, C602T, T652C, G720A	A650G	A + 652	KT808288.1
91	T6_3.1	“mixed” Ulagan	stallion	T495C, C602T, T703C, G720A, A740G	G604A	K2	KT808302.1
92	T6_4.1	“mixed” Ulagan	stallion	T495C, C602T, T703C, G720A, A740G	G585A, G604A	K2	KT808300.1
93	T6_4.2	“mixed” Ulagan	mare	T495C	–	D2	KT808291.1
94	T6_4.3	“mixed” Ulagan	stallion	T495C, A538G, C602T, C709T, G720A	G585A, A650G	I	KT808295.1

95	T6_5.1	“mixed” Ulagan	mare	T495C	–	D2	KT808292.1
96	T7_1.1	“mixed” Ulagan	stallion	T495C, A596G	–	D2 + 596	KT808293.1
97	T8_1.1	“mixed” Ulagan	mare	T495C, C602T, A667G, T703C, G720A	A597G	K3	KT808307.1
98	T8_1.2	“mixed” Ulagan	stallion	T495C, A538G, C602T, C709T, G720A	G585A, A650G	I	KT808296.1
99	T8_2.1	“mixed” Ulagan	mare	T494C, T495C, A496G, C534T, C602T, T603C, A649G, G720A	–	X2	KT808308.1
100	T8_2.2	“mixed” Ulagan	stallion	T495C, C542T, C602T, T632C, C635T, G666A, T703C, G720A	A597G, A650G	X3c1 + 632	KT808311.1
101	T8_2.3	“mixed” Ulagan	mare	T495C, C542T, C602T, T632C, C635T, G666A, T703C, G720A	A597G, A650G	X3c1 + 632	KT808312.1
102	T8_3.1	“mixed” Ulagan	stallion	T495C, C602T, T703C, G720A, A740G	G585A, G604A	K2	KT808301.1
103	T8_3.2	“mixed” Ulagan	mare	T495C, T526C, A540G, C602T, A649G, C718T, G720A	G585A	X4a	KT808314.1
104	T8_3.3	“mixed” Ulagan	mare	T495C, T526C, A540G, C602T, A649G, C718T, G720A	G585A	X4a	KT808315.1
105	T8_4.1	“mixed” Ulagan	stallion	T495C, T526C, A540G, C602T, A649G, C718T, G720A	G585A	X4a	KT808316.1
106	T8_4.2	“mixed” Ulagan	mare	T495C, C602T, T652C, G720A	A650G	A + 652	KT808289.1
107	T8_4.3	“mixed” Ulagan	–	T495C, C602T, T652C, G720A	A650G	A + 652	KT808290.1
108	T8_5.1	“mixed” Ulagan	–	T495C, C602T, A667G, T703C, G720A	A597G, G604A	K3	KT808306.1
109	T8_5.2	“mixed” Ulagan	mare	T494C, T495C, A496G, C534T, C602T, T603C, A649G, G720A	–	X2	KT808309.1
110	T8_5.3	“mixed” Ulagan	stallion	T495C, C602T, T703C, G720A, G726A, A740G	A597G, G604A	K2b	KT808303.1
111	KA1	“mixed” Kosh-Agach	mare	T495C, C602T, T617C, T659C, G720A	G585A	B1	KT808272.1
112	KA2	“mixed” Kosh-Agach	mare	T495C, T526C, A540G, C602T, A649G, C718T, G720A	G585A	X4a	KT808287.1
113	KA3	“mixed” Kosh-Agach	stallion	T494C, T495C, A496G, C534T, C602T, T603C, A649G, G720A	G585A, G604A	X2	KT808281.1
114	KA4	“mixed” Kosh-	mare	T494C, T495C, A496G, G521A, C534T,	G585A, G604A	X2 + 521	KT808283.1

		Agach		C602T, T603C, A649G, G720A			
115	KA5	“mixed” Kosh-Agach	stallion	T495C, T601C, C602T, G720A	–	F	KT808274.1
116	KA6	“mixed” Kosh-Agach	mare	T495C, C602T, A667G, T703C, G720A	–	K3	KT808277.1
117	KA7	“mixed” Kosh-Agach	mare	T494C, T495C, A496G, C534T, C602T, T603C, A649G, G720A	–	X2	KT808279.1
118	KA8	“mixed” Kosh-Agach	stallion	T495C, T526C, A540G, C602T, A649G, C718T, G720A	G585A	X4a	KT808286.1
119	KA9	“mixed” Kosh-Agach	stallion	T494C, T495C, A496G, C534T, C602T, T603C, A649G, G720A	G585A, G604A	X2	KT808282.1
120	KA10	“mixed” Kosh-Agach	mare	T494C, T495C, A496G, G521A, C534T, C602T, T603C, A649G, G720A	G585A, G604A	X2 + 521	KT808284.1
121	KA11	“mixed” Kosh-Agach	mare	T495C, C602T, T617C, T659C, G720A	–	B1	KT808270.1
122	KA12	“mixed” Kosh-Agach	mare	T495C, C602T, T617C, T659C, G720A	–	B1	KT808271.1
123	KA13	“mixed” Kosh-Agach	stallion	T495C, C602T, T617C, T659C, G720A	–	B1	KT808272.1
124	KA14	“mixed” Kosh-Agach	stallion	T494C, T495C, A496G, G521A, C534T, C602T, T603C, A649G, G720A	G585A, G604A	X2 + 521	KT808285.1
125	KA15	“mixed” Kosh-Agach	stallion	T495C, C602T, T703C, G720A, G726A, A740G	G585A, G604A	K2b	KT808276.1
126	KA16	“mixed” Kosh-Agach	stallion	T494C, T495C, A496G, C534T, C602T, T603C, A649G, G720A	–	X2	KT808280.1
127	KA17	“mixed” Kosh-Agach	stallion	T495C, C602T, T703C, G720A, A740G	G604A	K2	KT808275.1
128	KA18	“mixed” Kosh-Agach	stallion	T495C, C602T, A667G, T703C, G720A	A597G	K3	KT808278.1
