# Supplementary material for: Genetic History of the Altai Breed Horses: From Ancient Times to Modernity
Source: Genes (Basel). 2023 Jul 26;14(8):1523. doi: 10.3390/genes14081523 (PMC10454587; doi:10.3390/genes14081523)
Supplement: Supplementary file 1 [file genes-14-01523-s001.zip › Table S4. Squared cosines of the variables associated with the principal component analysis factors.pdf]

Table S4. Squared cosines of the variables associated with the principal component analysis factors.

Name of variable	Name of factor			
	F1	F2	F3	F4
A	**0.586**	0.242	0.128	0.044
B	0.268	**0.360**	0.162	0.210
C	**0.606**	0.374	0.019	0.001
D	**0.726**	0.194	0.012	0.068
D3	0.254	0.085	**0.653**	0.008
E	**0.606**	0.374	0.019	0.001
F	0.339	**0.472**	0.019	0.171
G	**0.606**	0.374	0.019	0.001
I	0.132	**0.396**	0.341	0.130
K	0.073	0.011	**0.842**	0.074
K3	0.300	0.001	0.137	**0.561**
X2	**0.428**	0.303	0.206	0.062
X3	**0.494**	0.166	0.293	0.046
X4	0.222	0.062	0.292	**0.424**
X5	**0.606**	0.374	0.019	0.001
X7	**0.606**	0.374	0.019	0.001

Values in bold indicate the largest variable squared cosine for each factor.
